# Clinical Outcome of Arthroscopic Bankart Repair and Remplissage in Recurrent Anterior Shoulder Dislocation in Manual Workers

**DOI:** 10.5704/MOJ.2507.013

**Published:** 2025-07

**Authors:** DK Sharma, P Shetty, N Kumar, A Kankane, K Anant, R Jamwal

**Affiliations:** Central Institute of Orthopaedics, Vardhman Mahavir Medical College and Safdarjung Hospital, New Delhi, India

**Keywords:** recurrent shoulder dislocation, arthroscopic Bankart repair, Remplissage, manual labour, engaging Hill-Sachs lesion

## Abstract

**Introduction:**

Recurrent anterior shoulder dislocation is particularly more common among occupations involving the frequent use of the upper limb above shoulder height such as manual labour. The present study aims to investigate outcomes of arthroscopic Bankart repair and Remplissage in manual workers as few studies have been undertaken in this specific subgroup. The arthroscopic Bankart repair and Remplissage in treatment of recurrent anterior shoulder dislocation in manual workers has reduced recurrence rates and improved patient satisfaction.

**Materials and methods:**

A total of 36 patients underwent arthroscopic Bankart with Remplissage for recurrent anterior shoulder dislocation, enrolled between February 2022 to December 2023 considering various inclusion and exclusion criteria. Patients were evaluated in post op period for range of motion, oxford shoulder instability score and visual analogue score for pain at intervals of 6 and 12 months.

**Results:**

All patients enrolled had soft tissue with bony Bankart lesion with mean of glenoid bone loss (%) was 11.75% (±3.15%) and all participants had engaging Hill-Sachs lesion. The mean pre-operative OSIS was 19.56 (±2.17) and mean VAS was 5.53 (±0.56). In the post op evaluation, there was no clinically significant decrease in range of motion with mean loss of external rotation of 3.86 (±1.44) at 12 months. The mean OSIS increased to 41.0 and mean VAS decreased to 2.36 at 12 months follow-up which was statistically significant. There was no incidence of dislocation in post op period of 12 months.

**Conclusion:**

Arthroscopic Bankart repair and Remplissage in recurrent anterior shoulder dislocation in manual workers demonstrates significant improvements in functional outcomes, minimal post-operative range of motion restrictions, and stable shoulders at the 12-month follow-up, highlighting the efficacy and safety of the procedure.

## Introduction

Anterior shoulder instability occurs when there's a soft tissue or bony injury in the shoulder, leading to the partial displacement or complete dislocation of the humeral head from the glenoid fossa. The lifetime risk of experiencing anterior shoulder instability is estimated to be between 1 to 2%^1^. Various risk factors contribute to recurrent anterior shoulder dislocation, including youth, engagement in high-impact contact sports, prior traumatic dislocation on the same side, the presence of Hill-Sachs or osseous Bankart lesions, inadequate rotator cuff or deltoid muscle function, and inherent ligamentous laxity^2-4^. Recurrent shoulder dislocation causes significant morbidity among patients associated with significant overhead activities such as among manual labourers^5,6^. Recurrent instability poses risks such as rotator cuff and axillary nerve injuries, along with progressive soft tissue and bony damage to the anterior glenoid. The long-term consequences of recurrent instability include the development of glenohumeral osteoarthritis, with 22.7% of patients experiencing clinically significant osteoarthritis in the glenohumeral joint at an average of 15 years post-dislocation^7^. Among the factors responsible for recurrent shoulder dislocation include Bankart and Hill-Sachs lesion^2^.

Arthroscopic Bankart repair involves reconstruction of Labrum employing multiple suture anchors for creation of anatomic barrier for stabilisation^8^. With advent of modern surgical techniques, It has proven to be minimally invasive, decreased intra-op time, post-op morbidity and blood loss, less complications and increased range of motion^9,10^. At present Arthroscopic repair using multiple suture anchors have placed it at par with open gold standard^11,12^. The collective occurrence rate of recurrent instability after this procedure stands at 15.3%, with variability spanning from 6.9% to 42% across different studies^13^.

Wolf and Pollack proposed capsulotenodesis of posterior capsule and infraspinatus tendon to fill the defect in the humeral head and named it as “Remplissage”. This method transforms the Hill–Sachs lesion from being within the joint to being extra articular, effectively preventing it from coming into contact with the anterior scapular glenoid^14^. This was shown to highly effective with minimal complication^15,16^. Some of the described pitfalls include decrease in postoperative range of motion especially in abduction and external rotation along with posterosuperior shoulder pain. The overall failure rate with combined arthroscopic Bankart and Remplissage was found to be 2-15%^17^.

The purpose of this study was to prospectively study clinical and functional outcomes after arthroscopic Bankart repair and Remplissage in cases of recurrent anterior shoulder dislocation among manual labourers. The outcome measures were range of motion, Oxford shoulder instability score (OSIS), visual analogue score (VAS) and re-dislocation rate. We hypothesised that the arthroscopic Bankart repair and Remplissage in treatment of recurrent anterior shoulder dislocation in manual workers has reduced recurrence rates and improved patient satisfaction.

## Materials And Methods

This was a prospective observational study on patients with history of manual labour who underwent arthroscopic Bankart repair Remplissage from April 2022 to February 2024. Permission was obtained from Institutional Ethics Committee (IEC).

The inclusion criterion was manual workers aged between 18 – 50 years with history of recurrent shoulder dislocation with soft tissue Bankart and bony Bankart lesion with up to 20% glenoid bone loss and engaging Hill Sachs identified on dynamic intra-operative assessment with minimum postoperative follow-up of 12 months. Patients meeting any of the following criteria are excluded from the study: a history of previous shoulder surgery, hyperlaxity of the shoulder joint, an inverted pear-shaped glenoid, multi-directional shoulder instability, uncontrolled or recurrent seizures, other shoulder abnormalities such as rotator cuff tears, or habitual shoulder dislocations.

At 95% confidence level and taking recurrence rate after arthroscopic Bankart repair and Remplissage in recurrent anterior shoulder dislocation subjects as 2% (Boileau *et al*^18^) and with an absolute error of 5%, the sample size estimated was 31. Considering 10% drop out rate, total sample size was 36. A total of 36 patients were enrolled in this study which was carried out in our institution.

Before the procedure, patients undergo a thorough history and clinical examination, followed by pre-operative 3D-CT and MRI scans, calculation of the Oxford shoulder instability score and visual Analog Score for pain, and assessment of the range of motion in the unaffected shoulder. As it is uncommon for patient with shoulder instability to have muscle wasting, loss of muscle bulk, if any, was noted. Patient subsequently undergoes pre-operative evaluation and admission a day prior to surgery. Patient demographics, surgical variables, and post-operative complications were documented. Demographic information encompassed age, sex, and the affected dominant shoulder (see Table I). Postoperative complications were defined as hematoma, infection, neurological injury, and any subsequent dislocations following arthroscopic repair. Patients underwent assessment during follow-up using the Oxford shoulder instability score and visual Analog Score for pain, and assessment of the range of motion in the affected shoulder at interval of 6 and 12 months.

**Table I:** Summary of basic details of participants.

Basic Details	Mean ± SD
Age (Years)	28.36 ± 6.08
Age	
18 - 30 years	26 (72.2%)
31 - 40 years	8 (22.2%)
41 - 50 years	2 (5.6%)
Gender	
Male	33 (91.7%)
Female	3 (8.3%)
Diagnosis side	
Right	22 (61.1%)
Left	14 (38.9%)
Duration since first dislocation (months)	16.97 ± 3.49
Occupational History	
Porters	21 (58.3%)
Carpenter	7 (19.4%)
Mason	5 (13.9%)
Painter	3 (8.3%)
Hand dominance	
Right	32 (88.9%)
Left	4 (11.1%)
Nature of Index dislocation	
Traumatic	36 (100.0%)
Total No. of dislocation	7.00 ± 1.69
Type of Bankart lesion	
Soft tissue with bony Bankart	36 (100.0%)
Amount of Glenoid bone loss(%)	11.75 ± 3.15
Nature of Hill-Sachs lesion	
Engaging	36 (100.0%)
Operated side	
Right	22 (61.1%)
Left	14 (38.9%)

**Fig. 1:**
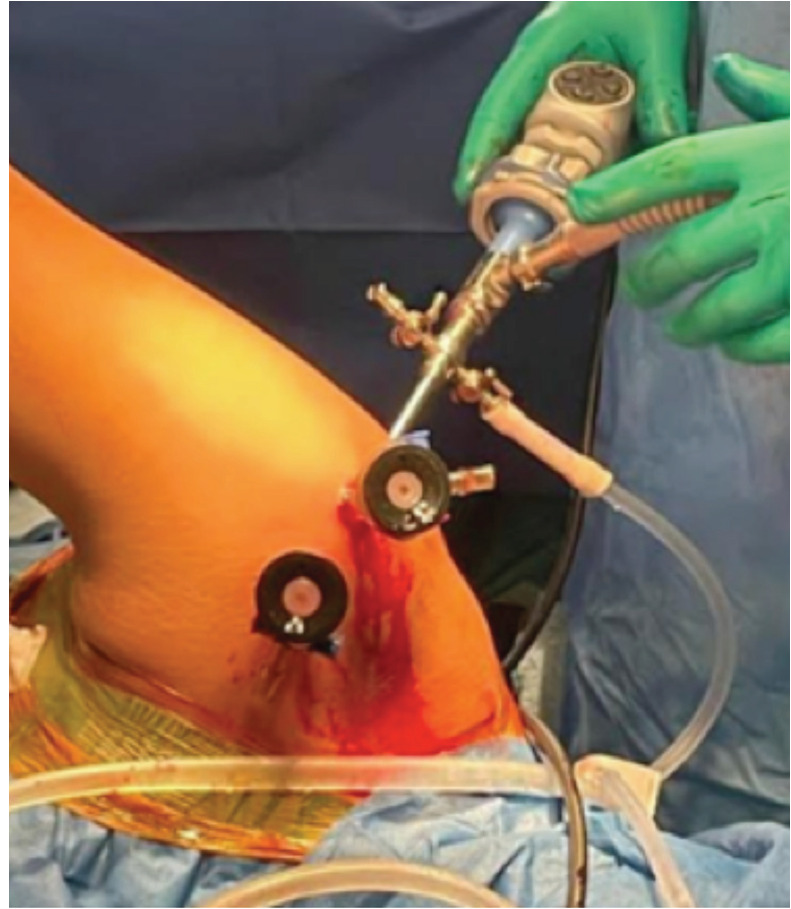
Operative set up showing standard posterior with two anterior portals.

All surgeries were performed by a single surgeon with ample experience in shoulder arthroscopy. All surgeries were conducted under hypotensive general anaesthesia in the lateral decubitus posture using a longitudinal and vertical 2-pulley traction system. The shoulder was set at 50° - 60° abduction, 20° - 30° forward flexion, and 20° posterior tilt to align the glenoid parallel to the ground. An evaluation under anaesthesia confirmed anterior shoulder instability. Entry into the glenohumeral joint was made through a primary posterior portal directly above the Hill-Sachs defect for visualisation and anchor placement. The surface landmark was 2cm inferior and lateral to the posterolateral tip of the acromion process. To address the labral tear, typically, two portals are created. An anterosuperior portal, primarily for visualisation and shuttle placement, is established at the anterior and lateral margin of the acromion, with the spinal needle entering the rotator interval at its superior margin and posterior to the long head of the biceps tendon. An anteroinferior portal, serving as a working portal for the inferior Labrum, is made in the rotator interval just proximal to the subscapularis tendon (Fig. 1). Diagnostic shoulder arthroscopy is performed, confirming an anteroinferior labral tear with glenoid bone loss. Dynamic intra-operative assessment confirmed a nature of Hill-Sachs lesion.

**Fig. 2:**
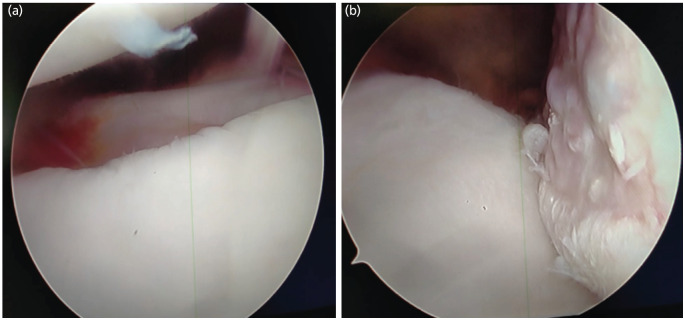
(a) Arthroscopic view showing anteroinferior labral tear. (b) Arthroscopic view of engaging “off-track” Hill-Sachs lesion over posterosuperolateral humeral head.

**Fig. 3:**
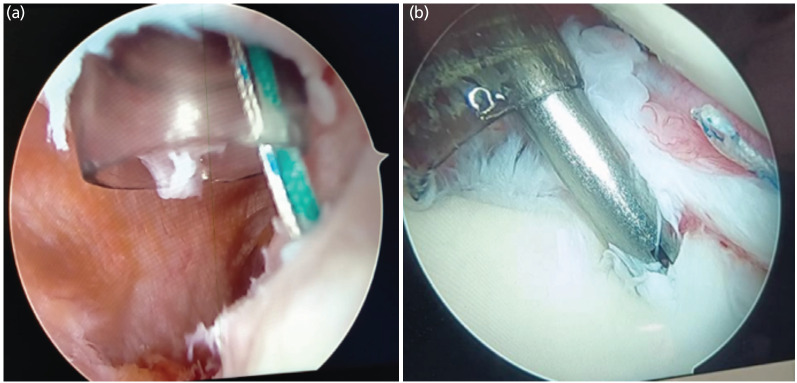
(a) A 5mm Wedge Anchor II with no.2 force fibre was placed over centre of Hill-Sachs lesion through posterior portal. (b) Placement of 3mm Wedge Anchor with no. 2 Force Fibre over anteroinferior Labrum.

**Fig. 4:**
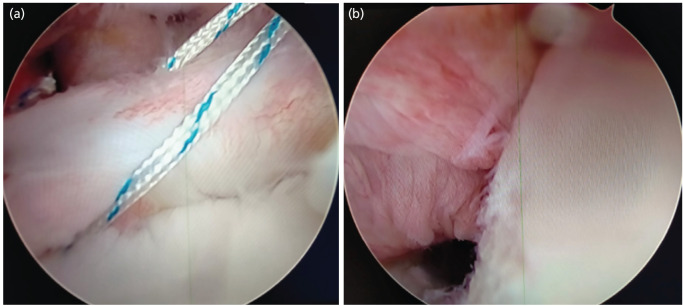
(a) Repair of anteroinferior Labrum and creation of Bumper effect. (b) View showing Infraspinatus tendon tenodesis to create Remplissage.

Glenoid preparation was performed using an arthroscopic rasp and shaver to facilitate adequate capsulolabral healing after repair from the posterior and anteroinferior portals (Fig. 2). The anteroinferior capsule with the inferior glenohumeral ligament and Labrum was mobilised with the help of an arthroscopic grasper and probe. After debridement with an arthroscopic shaver, a spinal needle was used to reach the Hill-Sachs lesion at its maximum depth while keeping it perpendicular to the lesion under vision from the posterior portal. A 5mm Wedge Anchor II with no.2 force fibre [Titanium, Stryker Corporation, Michigan, USA], a double-loaded suture anchor, was placed and gently tapped, followed by tightening until all the threads were buried. Using a penetrating grasper, one limb of each suture was retrieved through the posterior aspect of the capsule and the infraspinatus tendon (Fig. 3). Knotting of the suture was deferred at this point. A 3mm Wedge Anchor with no. 2 Force Fibre [Titanium, Stryker Corporation, Michigan, USA] suture anchor was placed at the 5.30, 2.0 and 4 o'clock positions, and Locking Mattress sutures were tied to create a Bumper effect at the anteroinferior Labrum Locking mattress knots of Remplissage were tied with the arm in neutral rotation (Fig. 4).

Post-operative rehabilitation protocol included immobilisation of the operated shoulder with an arm pouch for three weeks, followed by gradual introduction of exercises such as hand grip, elbow movements, and maintaining neutral rotation. Pendulum exercises and isometric cuff strengthening were introduced from the third to the sixth week, with range of motion exercises starting after six weeks and progressing to strengthening and proprioception exercises by the twelfth week. After assessing the strength and range of motion in comparison to normal shoulder, patients were encouraged to perform all painless activities including return to work assuming biological tissue healing after six months. Patients underwent post-operative clinical follow-ups at six weeks, three months, six months and one year. During these assessments, patients were evaluated for range of motion and any episodes of instability or dislocation. Shoulder function was assessed at six months and one year post-operatively using the Oxford shoulder instability score and Visual Analog Score for pain.

The collected data was entered in Microsoft Excel and then was analysed and statistically evaluated using SPSS-22 version. The mean and standard deviation (SD) for the quantitative functional scores were calculated.

Non-parametric tests such as Friedman test were used to make statistical inference of range of motion as data were not normally distributed. Friedman test was used to explore whether the Range of motion i.e., forward flexion, abduction, internal rotation and external rotation changed significantly over time. The data regarding the change in Oxford shoulder instability score was normally distributed. Hence, ANOVA was used to explore whether the OSIS changed significantly over time. Friedman test was used to explore whether the VAS Score changed significantly over time as the data was not normally distributed. P<0.001 was considered to be statistically significant.

## Results

After examining the inclusion and exclusion criteria, 36 patients were enrolled in the study. All patients underwent primary surgery (100%), and there were no revision surgeries in our study. All the patients were followed-up as per the protocol for the period of 12 months. The average age of the patients at surgery was 28.36 ± 6.08 years. Among the participants, 33 of the 36 (91.7%) were males and 3 of the 36 (8.3%) were females. Among the side operated, 61.1% (22 of 36) were operated in the right side and 38.9% (14 of 36) were operated in the left side. 21 (58.3%) of the participants were porters, 7 (19.4%) of the participants were carpenters, 5 (13.9%) of the participants were masons and 3 (8.3%) of the participants had occupational history of painter. All the patients included in the study had history of traumatic index dislocation. The mean of total time since index dislocation was 16.97 ± 3.49 months. The mean of total number of dislocations among participants was 7.00 ± 1.69. All 36 (100.0%) participants exhibited bony with soft tissue Bankart lesion on diagnostic imaging. The mean amount of glenoid bone loss (%) was 11.75 ± 3.15%. Additionally, all 36 (100.0%) participants were noted to have engaging Hill-Sach lesion on dynamic intra-operative evaluation. These demographic data have been demonstrated in a Table I.

The pre-operative range of motion was evaluated on non-affected shoulder followed measurement of ROM in the operated shoulder at subsequent follow-up at 6 months and 12 months (Table II). On evaluation of pre-operative abduction of shoulder, the mean (SD) of abduction (preoperative) was 169.92° (±3.28), mean forward flexion was 169. A total of 58° (±3.67), mean external rotation was 68.78° (±2.34) and mean internal rotation was 69.06° (±2.22). None of the patients had significant muscle wasting pre-operatively.

**Table II:** Summary of range of motion.

Range of Motion	Mean ± SD
Pre-operative	
Abduction	169.92 ± 3.28
Forward flexion	169.58 ± 3.67
External rotation	68.78 ± 2.34
Internal rotation	69.06 ± 2.22
Post-operative at 6 months	
Abduction	167.58 ± 3.18
Forward flexion	167.78 ± 3.30
External rotation	66.14 ± 2.10
Internal rotation	66.58 ± 2.31
Post-operative at 12 months	
Abduction	168.69 ± 3.38
Forward flexion	168.36 ± 3.42
External rotation	64.92 ± 1.81
Internal rotation	68.22 ± 2.44

The functional assessment was performed in pre-operative period with determination of Oxford Shoulder Instability Score and Visual analogue score for pain. In the preoperative period, the mean Oxford Shoulder Instability Score (OSIS) was 19.56 (SD = 2.17), while the mean Visual Analog Scale (VAS) score was 5.53 (SD = 0.56) (Table III).

**Table III:** Summary of Oxford shoulder instability score (OSIS) and visual analogue score (VAS) for pain.

Score	Mean ± SD
Oxford shoulder instability score	
Pre-operative	19.56 ± 2.17
Post-operative at 6 months	41.00 ± 1.71
Post-operative at 12 months	41.00 ± 1.53
Visual Analogue Score for pain	
Pre-operative	5.53 ± 0.56
Post-operative at 6 months	2.36 ± 0.49
Post-operative at 12 months	2.36 ± 0.49

The mean forward flexion decreased from a maximum of 169.58 at the pre-operative timepoint to a minimum of 167.78 at 6 Months, then increased to 168.36 at 12 Months, with no clinically significant reduction observed, while external rotation decreased from a maximum of 68.78 at preoperative to a minimum of 64.92 at 12 Months, although statistically significant (P<0.001), it was not clinically significant, similarly, internal rotation decreased from a maximum of 69.06 at pre-operative to a minimum of 66.58 at 6 Months, then increased to 68.22 at 12 Months, with no clinically significant change, and abduction decreased from a maximum of 169.92 at pre-operative to a minimum of 167.5 at 6 Months, then increased to 168.69 at 12 Months, without clinical significance. The mean loss of external rotation at 6 months was 2.64 ± 1.55 and at 12 months was 3.86 ± 1.44.

The mean OSIS increased from a minimum of 19.56 ± 2.17 at the pre-operative timepoint to a maximum of 41.00 ± 1.53 at the 12 Months timepoint. This change was statistically significant (P<0.001). The mean VAS Score decreased from a maximum of 5.53 ± 0.56 at the pre-operative timepoint to a minimum of 2.36 ± 0.49 at the 12 Months timepoint. This change was statistically significant (P<0.001).

All the patients had no events of dislocation until 12 months of follow-up with no significant complication. All patients returned to their pre-operative occupation. 

## Discussion

In developing countries with higher occupational disease burden, recurrent anterior shoulder dislocation is of a particular concern, considering the morbidity it can result with association with loss of productivity in addition to financial and mental distress^19^. Manual laborers included in study had history of wide variety of overhead activities like lifting of objects over back and head, working with ceiling and hammering. With wide array of proposed surgical interventions, Arthroscopic Bankart repair with Remplissage along with bony procedures such as Latarjet procedure find a remarkable mention. The benefits conferred by arthroscopic Bankart repair with Remplissage include enhanced shoulder stability, reduced recurrence rates, preserved range of motion, facilitated faster recovery, and

has favourable outcomes in terms of stability and patient satisfaction^20^.

Incorporating the infraspinatus transfer into a traditional Bankart repair, when suitable, offers two significant advantages. Firstly, the posterior capsulotenodesis relocates the Hill-Sachs defect outside the joint, preventing its engagement with the anteroinferior glenoid rim during abduction and external rotation. Secondly, the Remplissage technique hinders anterior translation of the humeral head by creating a tethering effect^11,20^.

Age has a significant bearing on the incidence as well as therapeutic outcome of recurrent anterior shoulder dislocation. Among 36 patients who were enrolled in the study, mean age was 28.36 year which was similar to study conducted by Pathak *et al*^17^, Boileu *et al*^18^, Metz *et al*^20^ in the range was 19-47 years. The distribution of population was 72.2% of the participants had age: 18 –30 years, 22.2% of the participants had age: 31 – 40 years, and 5.6% of the participants had age: 41-50 years. There was no statistically significant correlation between glenoid bone loss and Oxford shoulder instability score (OSIS) and VAS Score in preoperative similar to study by Carrazzone *et al*^21^.

In our study, we found that there was no significant difference between the various groups in terms of distribution of diagnosis side (χ^2^ = 0.234, p = 0.634). This was in concurrence with meta-analysis performed by Zhang *et al*^13^ which concluded that there is no evidence to suggest that dominant side is an important risk factor for recurrent instability following a Bankart procedure.

All the patients enrolled in the study were having history of recurrent dislocation following traumatic primary dislocation. All the patients enrolled had soft tissue with bony Bankart lesion as diagnosed by MRI and 3D CT of shoulder. Among the patients enrolled in the study, The mean of amount of glenoid bone loss (%) was 11.75 (±3.15). The amount of glenoid bone loss (%) ranged from 5 - 19. This was similar to study conducted by Paul *et al*^22^ which had average glenoid bone loss of 11%. In a study conducted by Miyamoto *et al*^4^ among 18 patients, observed that amount of glenoid bone loss among patients with sub critical glenoid bone loss (<25%) was not correlated with outcomes.

After comparing abduction changes post-operatively to the contralateral shoulder's pre-operative status, we found a decrease from 169.92 to 167.58 at 6 months, followed by an increase to 168.69 at 12 months. This change did not reach statistical significance (repeated measures ANOVA: F=3.0, p=0.052). Upon comparing abduction changes postoperatively to the contralateral shoulder's pre-operative status, we found a decrease from 169.92 to 167.58 at 6 months, followed by an increase to 168.69 at 12 months. This change did not reach statistical significance (repeated measures ANOVA: F=3.0, p=0.052), indicating a lack of clinically significant difference.

Although the Remplissage with Bankart repair procedure has had increasing acceptance, there are still some concerns about post-operative stiffness and especially external rotation loss. However, the biomechanical study of Argintar *et al*^23^ showed that the Bankart repair restored all ER to normal values at 0° and 60° abduction, and the addition of the Remplissage procedure did not significantly alter the overall ROM and the anteroposterior translation. On observing the change of external flexion in post-operative period after initial examination as described above, we noted that the mean ROM: External Rotation decreased from a maximum of 68.78 at the pre-operative timepoint to a minimum of 64.92 at the 12 Months timepoint. This change was statistically significant (Friedman Test: χ^2^ = 60.9, p = <0.001). The mean loss of external rotation at 6 months was -2.64 ± 1.55 and at 12 months was -3.86 ± 1.44. The range of loss of motion at 12 months was -7.0 to -1.0. This loss of external rotation was similar to observed in Pathak *et al*^17^, Boileau *et a*^l18^ and Zhu *et al*^24^. This loss of external rotation was not found to significantly effect activities of daily living.

On evaluation of Oxford shoulder instability score (OSIS), We noted that the mean OSIS increased from a minimum of 19.56 at the pre-operative timepoint to a maximum of 41.00 at the 12 months timepoint. This change was statistically significant (repeated measures ANOVA: F=365.9, p = <0.001). This was similar to study conducted by Pathak *et al*^17^ who observed that the average Oxford Shoulder Instability Score for the involved shoulder increased from 21.95 ± 3.09 pre-operatively to 41.29 ± 2.31 (P <.001) postoperatively.

On evaluation of VAS Score, the mean VAS Score decreased from a maximum of 5.53 at the pre-operative timepoint to a minimum of 2.36 at the 12 months timepoint. This change was statistically significant (Friedman Test: χ^2^ = 72.0, p = <0.001). In a study conducted by Nourissat *et al*^16^, observed that 1/3rd of the patients continued to have shoulder pain. It was postulated to be likely due to non-healing of Hill Sach’s lesion^18^, impingement between the posterior Labrum and the new footprint of the posterior cuff^16^, capsulomyodesis rather than capsulotenodesis in Remplissage^25^.

It was observed that there was no statistically significant correlation between duration since first dislocation and OSIS (pre-operative) (rho = 0.24, p = 0.157). Similar findings was observed in a study conducted by Yamak *et al*^26^ in 2023.

It was also observed that pre-operative VAS Score had statistically significant negative correlation with change in VAS Score at 6 months and 12 months. This implies that patients with higher VAS score had less change in VAS score in post-operative period of Arthroscopic Bankart repair and Remplissage.

Shoulder dislocation and subluxations were considered as failure of the treatment. There was no incidence of failure of the procedure among 36 patients who underwent the procedure in the follow-up period of 12 months. There were no complications reported among 36 patients. All 100% patients were found to be having stable shoulder at time of last follow at 12 months. As per the available literature, failure rate in the long-term follow-up after Arthroscopic Bankart repair and Remplissage found to be ranging from 2 to 15%. A study conducted by Miyamoto *et al*^4^ had similar outcome at end of 12 months of follow-up. A study conducted by Boileau *et al*^18^ had recurrence rate of 2% at end of 24 months.

The study's strengths include its exploration of functional outcomes of arthroscopic Bankart repair and Remplissage among manual laborers, a demographic with limited literature despite recurrent anterior shoulder dislocation risks from high overhead activities, conducted in a tertiary care centre with a high patient load from lower and middle socioeconomic groups, facilitating potential replication without significant economic burden, performed by experienced surgeons with thorough post-operative rehabilitation, and maintaining strict follow-up protocols for accurate data collection. However, limitations include a small sample size, observational cohort design possibly leading to selection bias, short 12-month follow-up duration without observation of redislocation or subluxation, lack of evaluation using other functional outcome measures such as the ROWE score and ASES Score, indicating the need for multicentric studies with larger sample sizes for community application.

## Conclusion

Our study on "Arthroscopic Bankart Repair and Remplissage in Recurrent Anterior Shoulder Dislocation in Manual Workers" demonstrated significant improvements in functional outcomes, minimal post-operative range of motion restrictions, and stable shoulders at the 12-month follow-up, highlighting the efficacy and safety of the procedure.
